# Carer involvement in the transition from inpatient to community mental healthcare: Experiences of stakeholders

**DOI:** 10.1192/j.eurpsy.2022.1563

**Published:** 2022-09-01

**Authors:** A. Kouroupa, E. Petkari, D. Giacco

**Affiliations:** 1 University College London, Psychiatry, London, United Kingdom; 2 Universidad Internacional de La Rioja, Health, Logroño, Spain; 3 University of Warwick, Wms - Health Sciences, Warwick, United Kingdom

**Keywords:** inpatient, carer involvement, service transition, community care

## Abstract

**Introduction:**

The involvement of informal carers (family and friends) in the care of people with severe mental illness (SMI) contributes to positive clinical outcomes, such as relapse prevention and symptom reduction. To date, the care pathway between inpatient and community care is not clearly defined impeding the smooth transition for patients, whilst carers are still barely involved in shared decision-making processes.

**Objectives:**

To investigate the views and experiences of patients with SMI, carers and clinicians regarding the transition from inpatient to community mental health services.

**Methods:**

Four mixed focus groups were conducted with individuals with SMI (n=12), carers (n=10) and clinicians (n=9) across four different mental health catchment areas in England. Participants discussed their experiences and provided their views on facilitators, barriers and solutions for carer involvement during the transition between mental health services. Data were analysed using thematic analysis.

**Results:**

All stakeholders highlighted that factors that impede carer involvement are related to: confidentiality issues, unmet (structural and organisational) needs, and carer expectations. Patients with SMI, carers and clinicians agreed that carer involvement can be improved by providing psychoeducation to carers and training to staff, having accessible and transparent clinical procedures, and allocating specialised staff to carers.

**Conclusions:**

The study findings emphasise that carer involvement is still overlooked, particularly when adults with SMI transition between services. The results provide guidance for practice emphasising the need for systematic involvement of carers across inpatient care, and for future research proposing effective ways of maximising carer involvement in mental health care.

**Disclosure:**

No significant relationships.

